# Maternal and Embryonic Stress Influence Offspring Behavior in the Cuttlefish *Sepia officinalis*

**DOI:** 10.3389/fphys.2017.00981

**Published:** 2017-12-01

**Authors:** Caitlin E. O'Brien, Christelle Jozet-Alves, Nawel Mezrai, Cécile Bellanger, Anne-Sophie Darmaillacq, Ludovic Dickel

**Affiliations:** Normandie Univ., UNICAEN, Rennes 1 Univ., UR1, CNRS, UMR 6552 ETHOS, Caen, France

**Keywords:** body patterning, predation, visual lateralization, activity, threat response

## Abstract

Stress experienced during prenatal development—either applied to reproducing females (maternal stress), directly to developing offspring (embryonic stress) or in combination—is associated with a range of post-natal behavioral effects in numerous organisms. We conducted an experiment to discern if maternal and embryonic stressors affect the behavior of hatchlings of the cuttlefish *Sepia officinalis*, a species with features that allow for the examination of these stress types in isolation. Separating the impact of stress transmitted through the mother vs. stress experienced by the embryo itself will help clarify the behavioral findings in viviparous species for which it is impossible to disentangle these effects. We also compared the effect of a naturally-occurring (predator cue) and an “artificial” (bright, randomly-occurring LED light) embryonic stressor. This allowed us to test the hypothesis that a threat commonly faced by a species (natural threat) would be met with a genetically-programmed and adaptive response while a novel one would confound innate defense mechanisms and lead to maladaptive effects. We found that the maternal stressor was associated with significant differences in body patterning and activity patterns. By contrast, embryonic exposure to stressors increased the proportion of individuals that pursued prey. From these results, it appears that in cuttlefish, maternal and embryonic stressors affect different post-natal behavior in offspring. In addition, the effect of the artificial stressor suggests that organisms can sometimes react adaptively to a stressor even if it is not one that has been encountered during the evolutionary history of the species.

## Introduction

Stress responses occur in reaction to any external or anticipated threat. In response to a predator, for instance, an animal may increase its metabolism and divert resources to its muscles and away from less critical functions like digestion and foraging behavior—the “fight or flight” stress response (Cannon, 1939). Other kinds of stressors will induce different reactions. In response to food scarcity, for instance, an animal may have the opposite reaction, prioritizing digestive processes to extract the maximum amount of energy from food items and even undertaking risky foraging behavior (Wang et al., 2006). While stress responses have presumably evolved to increase survival in the face of an immediate stressor, there is an increasing awareness that stress responses come with a host of negative fitness consequences. This often depends on whether the stressor causing the response is acute or chronic: A short, single experience of a stressor (e.g., a single encounter with a predator) often produces a short-term, adaptive response while long-term or repeated exposure to stressors (e.g., prolonged food shortage) can have lasting negative impacts on fitness (Jones, 1996; Miller et al., 2007). These costs come from the energetic tradeoffs involved in maintaining the response or in the form of missed opportunities (e.g., lost foraging time, mating opportunities). Chronic and repeated stressors are often associated with reductions in immune function, the advent of various diseases, negative impacts on psychological health and disruptions to normal biological functions (e.g., Katz et al., 1981; Miller et al., 2007; Favreau-Peigné et al., 2014). Thus, understanding the underlying causes and effects of stress responses has implications for medicine, psychology and developmental biology, and is studied in a number of animal models.

The long-term effects of stress that occurs during the embryonic development of an organism are known to be especially significant. Research in a number of vertebrate taxa demonstrates that stress responses in reproducing females can have a strong impact on the behavior of her offspring. In some cases, such stress may serve as an indicator of prospective environment, prompting adaptive changes to the offspring phenotype that help it cope with future challenges. Stress responses can also be associated with reduced offspring fitness; normal developmental processes can be disrupted and the animal may be more susceptible to disease (Gluckman and Hanson, 2004). While the effects of prenatal stress have been relatively well-documented in a number of taxa, it is often unclear if effects observed are the direct result of a stress response in the offspring or a maternally–transmitted effect. One potential mechanism for prenatal stress effects in offspring is the transfer of “stress hormones” (e.g., glucocorticoids, catecholamines) from mother to developing embryo. Such hormones are secreted by animals in response to stressors and affect physiology, behavior and metabolism. Their transfer to offspring via the placenta or egg yolk could explain many of the alterations to offspring phenotype that are sometimes observed (Hayward and Wingfield, 2004; Groothuis et al., 2005; Weinstock, 2008).

Alternatively or in parallel, embryos could be experiencing stressors directly and generating their own stress responses. Where most authors use the term “prenatal stress” to refer to an offspring's response to any stressor experienced during embryonic development, we distinguish between effects of stressors applied to the mother (“maternal stress”) and those applied to the offspring themselves (“embryonic stress”). Investigations of stressors applied directly to developing embryos are much less numerous than studies of maternally-applied stress, largely for logistical reasons. By necessity, prenatal stressors must be applied to pregnant or brooding females in many behavioral models, since their embryos develop viviparously or ovoviviparously. Moreover, it has only recently become widely recognized that the embryos of many species are able to perceive and react to stimuli in the surrounding environment, and that this sensory input could provide essential information to prepare for challenges in the postnatal environment (e.g., Mathis et al., 2008). One way to gauge the relative contributions of maternal and embryonic stress responses is to compare their effects in experimental isolation using animal models that are oviparous and autonomous at birth (e.g., many fish, amphibians, precocial birds, and invertebrates). For example, experiments have demonstrated that rainbow trout eggs exposed to stress hormones (comparable to what a stressed mother might produce) result in offspring that are more fearful 5 months after hatching than control animals, although no differences were seen at 2 months (Colson et al., 2015). Likewise, when eggs of the same species were isolated from their mothers and subjected to conspecific alarms cues they demonstrated greater behavioral plasticity than non-stressed controls (Poisson et al., 2017). Therefore, it seems that both maternal and embryonic stressors affect behavior in this species. However, experiments with another species of trout failed to show any differences induced by prenatal stress, suggesting that susceptibility to prenatal stress is not universal across this subfamily (Ghio et al., 2016). By comparing these three studies, we can see that stress effects differ depending on stress type, species, context and age, a finding that likely holds true for other groups as well.

Despite their potential as good study models, there is an unfortunate lack of work with invertebrates, perhaps because invertebrates are sometimes considered unsophisticated and thus unworthy of behavioral study, and because experiments are complicated by the existence of larval phases in many species. The cuttlefish *Sepia officinalis* (Linnaeus, 1758) has neither of these issues. Like other coleoid cephalopods, it is neurologically and behaviorally sophisticated but unlike other coleoids and invertebrates, it has no pelagic larval stage, settling directly on the bottom after hatching (Hanlon and Messenger, 1998). Even more importantly for a potential model for the study of prenatal stress, this species is known to perceive and learn from within the egg (Romagny et al., 2012). A number of embryonic influences have already been identified in cuttlefish. For instance, embryos can develop post-hatching prey preferences and behavioral asymmetries from visual or odor cues (Darmaillacq et al., 2008; Jozet-Alves and Hebert, 2012) and habituate to repeated sensory stimuli, such as light, odor and tactile cues (Romagny et al., 2012). Documenting the effects of maternal and embryonic stress in this species may elucidate general principals about how animal offspring are affected by different types of stress, or indicate that the impact differs according to phylum. In addition, a better understanding of the effects of maternal and embryonic stress in *S. officinalis* would have direct implications for the welfare of cephalopods in aquaculture, laboratories and aquaria. This is important as cephalopods are increasingly recognized as advanced organisms capable of pain and suffering and were recently added to the list of protected animal groups covered by European welfare legislation (Directive 2010/63/EU).

In order to determine whether prenatal stress affects cuttlefish behavior, we subjected reproducing female cuttlefish and their eggs to stressful stimuli. Our primary goal was to determine if female cuttlefish transmit stress effects to their offspring. To this end, we compared the offspring of “unstressed” and “stressed” captive females. We also included a group of “wild” eggs in order to assess whether captivity during egg-laying exerts any effects. Our secondary goal was to assess the relevance of stressor type to offspring. We tested the hypothesis that stress responses depend on stress type, particularly how “familiar” it is to the species. We predicted that a naturally-occurring stressor like odor cues from a co-occurring predator species would elicit an adaptive anti-predator response genetically programmed by natural selection. In contrast, we predicted that an artificial stressor would confound innate defense mechanisms and provoke behavioral responses with largely negative effects on fitness. We tested this hypothesis by comparing the effects of an artificial stressor (randomized bouts of bright LED light) to a naturally-occurring one (predator odor) applied to developing embryos. Experiments had already demonstrated that prenatal exposure to predator odor affect the post-natal behavioral lateralization of cuttlefish (Jozet-Alves and Hebert, 2012). LED light was selected as the artificial stressor since it can be detected by late-stage embryos (Romagny et al., 2012) and is likely to be present in aquacultural facilities and laboratories. Immediately after hatching, the offspring from each of these stress groups were tested in a battery of behavioral tests. These tests were chosen to assess a wide range of behaviors thought to be crucial to survival in the wild: body patterning, predation ability, brain lateralization, baseline activity and activity in response to an imminent threat. Behavior was tested during the first 10 days after hatching as this is thought to be the time of highest mortality in the lifecycle of cuttlefish (Bloor et al., 2013).

## Methods

**Figure 1:**
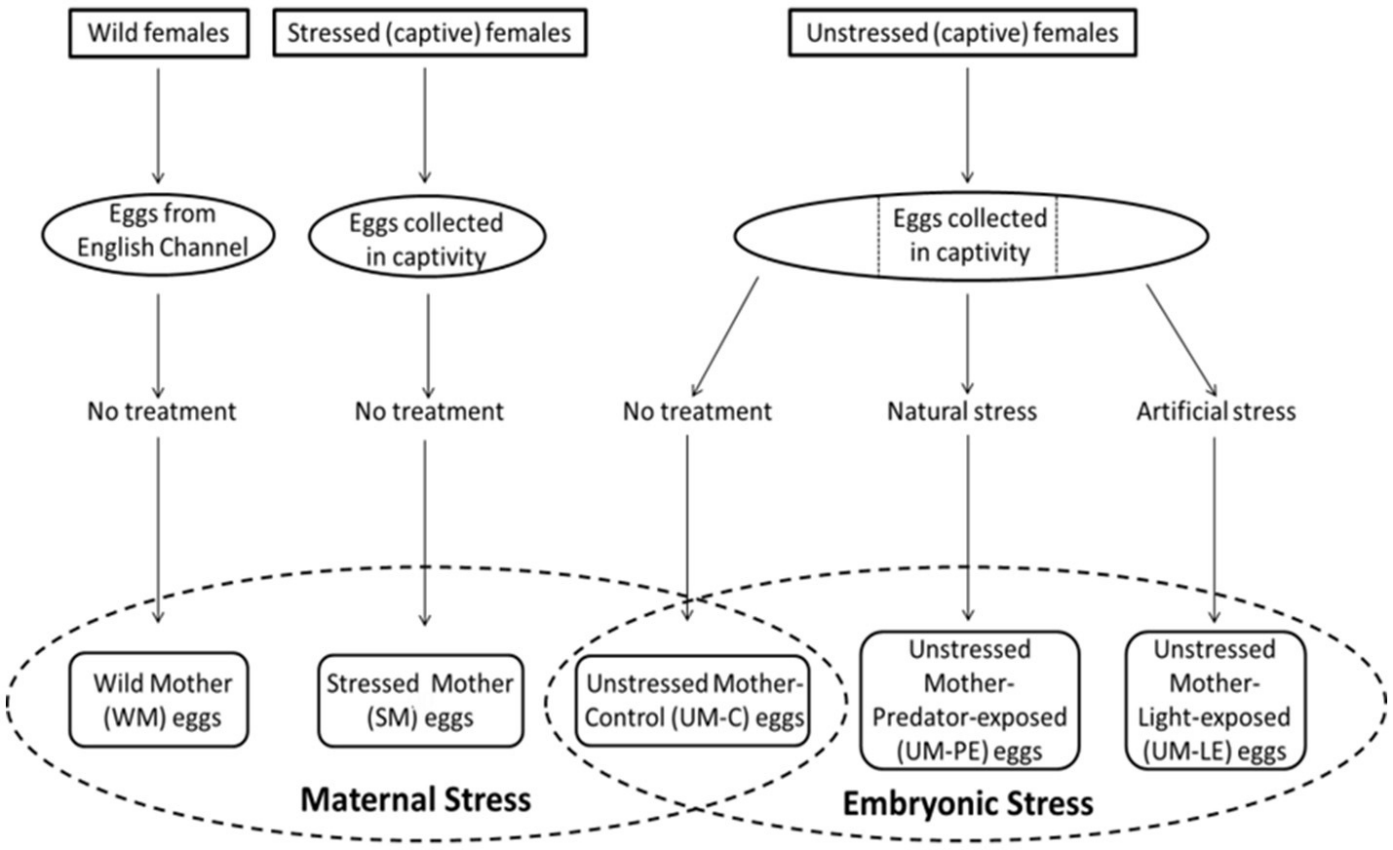
Schematic representation of the experimental design.

Two different experiments were conducted, one testing for the potential transfer of the effects of captivity or stress from reproducing females to their offspring, and a second exploring the impact of stressors applied directly to developing embryos. In the first experiment, we exposed spawning female cuttlefish to daily removal from the water. We then compared the behavior of their offspring (SM) to that of offspring of a group of captive but unstressed mother controls (UM-C). We also compared both of these groups to offspring from naturally-spawned eggs collected from the wild (WM). While the maternal experience of these eggs was unknown and uncontrolled, their inclusion gives a sense of the effects of maternal capture and captivity (Figure 1).

In the second experiment testing embryonic stress, we subdivided eggs from the unstressed control mothers into three groups in order to investigate the effects of stimuli applied directly to embryos. We applied two kinds of stressors: a naturally-occurring stressor consisting of odor cues from common predatory fish (UM-PE) and an artificial stressor consisting of high intensity LED light timed randomly and unpredictably throughout the day and night (UM-LE). These two groups were compared to the unstressed mother control (UM-C) group used in the maternal stress comparisons (Figure 1).

After hatching, the effects of prenatal stress treatments on offspring were assessed with a battery of tests covering various aspects of the cuttlefish behavioral repertoire, including body patterning, visual lateralization, predation, activity patterns and fear response. These tests allowed us to make a broad assessment as to whether stressors affect offspring behavior and to make general comparisons between embryonic and maternal stress and between a natural and an artificial stressor. We predicted that the direct experience of an embryonic stressor would have a stronger effect on offspring behavior than maternal stress, which consists of information that must be transmitted indirectly to offspring through the mother. We also expected that cuttlefish would have evolved adaptive responses to the natural embryonic stressor (predator odor), but would demonstrate inappropriate and likely maladaptive responses to the artificial stressor since its response to this stimulus could not have been shaped by natural selection.

### Animal collection and housing

#### Adult females

Cuttlefish traps were set off the coast of France in the English Channel. Thirty seven adult female cuttlefish (*S. officinalis*) were captured between May and June, 2015 and 28 were captured in May, 2016 and transported to the Centre de Recherches en Environnement Côtier (CREC, marine station of the University of Caen, Luc-sur-Mer, France). These females were mated with males and then placed in treatment tanks in a semi-open flow-through seawater system (15 ± 1°C) under a 16:8 h light/dark cycle.

Captured females were split randomly into two groups, and eggs collected from them were designated as “unstressed mother—control” and “stressed mother.” The females designated as unstressed mothers (six in 2015 and 12 in 2016) were maintained in large (1,000 L), round tanks enriched with stones, plastic algae, floating objects and plenty of shaded area. In 2015, these females were housed in these tanks singly, but in 2016, the capture of two dozen cuttlefish on a single day necessitated housing in groups of three. Those females assigned to be in the stressed mothers group were isolated in bare tanks (65 L) with a water depth of 19 cm and subjected to randomized 10-s removals from the water three times a day using a specially-made mesh platform. Eggs spawned by these stressed captive females after at least 1 week in these conditions were collected. Four unstressed mothers and four stressed mothers spawned between May 15 and June 9, 2015 and 11 unstressed mothers and eight stressed mothers spawned between May 14 and 29, 2016.

#### Eggs and hatchlings

Wild mother eggs (WM) were collected by SCUBA divers from pre-placed tethers in the English Channel (49°19.667N-0°18.767W) in June, 2015 from a depth of 13.7 m. These, along with eggs collected from unstressed and stressed mothers in captivity, were moved to floating trays in 65L tanks (80 × 60 × 40 cm) after 8 h of steady temperature habituation (from 15° to 20°C). These were housed in a darkened room with exposure to the natural light cycle and supplied with seawater from a gently flowing open system and aerated by an airstone. A randomly-selected third of the control mother eggs, designated as controls (UM-C), along with WM and stressed mother SM eggs, were not treated any further. The other two thirds of the control mother eggs were divided randomly into predator-exposed (UM-PE) and light-exposed (UM-LE) groups. Three sea bass (Linnaeus, 1758; Dicentrarchus labrax; total length = 25–30 cm) were housed with UM-PE eggs, separated by a mesh barrier that allowed the eggs chemosensory and visual exposure to the predatory fish. Light-exposed eggs experienced strong LED illumination (20.7klux, approximately 10 cm from surface of water) for 90 min a day (six randomly-timed periods of 15 min). All eggs were gently agitated once a day to remove detritus and discourage parasite growth.

Hatchlings were recorded and collected at 08:00 each morning between June 29 and August 5, 2015, and July 2–24, 2016, and then transferred to a new tank to remove them from any further exposure to the stress treatments. Between experiments, hatchlings and juveniles were maintained in individually-labeled compartments to preserve identity. These compartments were situated in an aerated open seawater system (19–23°C) with a water depth of 7 cm. Sex determination was not possible at this age. All hatchlings born on a single day comprised a daily cohort. A total of 22 cohorts (numbering up to 12 individuals each) were hatched and tested daily between July and August. In 2015, after the predation experiment on Day 4, individuals were fed a single shrimp (Crangon crangon; Linnaeus, 1758) per day. In 2016, hatchlings were fed *ad libitum* starting on Day 4.

### Behavioral experiments

**Figure 2:**
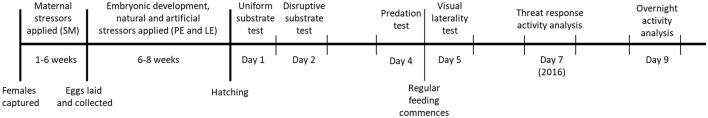
Timeline of stress treatments and behavioral tests. All tests except for the threat response activity analysis occurred in 2015.

Following the 2 months of prenatal stress treatments described in the previous section, the resulting offspring were subjected to a battery of tests conducted during the first 10 days after hatching (Figure 2). These behavioral tests were selected to determine whether the stress treatments had affected certain key aspects of the behavioral phenotype—body patterning, predation ability, brain lateralization, activity level and response to a threat. The data resulting from these tests were analyzed in R, GraphPad (Prism®) and StatXact®7 (Cytel Inc.). All *p*-values are two-tailed and alpha was set at 0.05.

#### Body patterning

In 2015, on the day of hatching (Day 1), between 9:00 and 10:30, up to 12 cuttlefish at a time were placed in randomized order in small uniform gray (“uniform background”) circular compartments with slanted sides to minimize shadows (radius = 2.9 cm bottom, 3.35 cm top, length of sides = 2.5 cm; mean gray value = 101 ± 3.9) under white LED light (0.63 to 0.88 klux) and photographed at 0, 5, 15, and 30 min after placement on the background with a Panasonic HDC-SD60 camera. On Day 2, between 10:30-12:00, cuttlefish order was re-randomized and each was photographed four times (0, 5, 15, and 30 min after placement) against a checkered pattern (“disruptive background”). The check size of the disruptive background was selected to be approximately the size of a hatchling's main body-patterning component, the dorsal mantle square (3 × 3 mm), since previous studies have shown that this usually elicits a disruptive pattern in cuttlefish (Chiao et al., 2015).

ImageJ was used to assess the heterogeneity index (HI), a measure of body pattern disruptiveness, of individuals from the photographs. By selecting the outline of the mantle by hand and measuring the “standard deviation,” HI was calculated from the standard deviation between the mean gray values of every individual pixel (x) comprising the dorsal mantle ($\bar{x}$), and the total number of pixels (N) selected, with higher values indicating higher overall disruptiveness of body patterning (see methodological description in Di Poi et al., 2014).

(1) $$\begin{array}{r} \text{HI}=\sqrt{\frac{1}{N}\sum {\left(x-\bar{x}\right)}^{2}} \end{array}$$

Only photographs in which cuttlefish had settled and remained motionless were used for these measurements. Because there was little variation over time in individuals' HI, the values from the four time points were averaged and used to calculate group means for each background type. In total, 55 WM, 41 UM-C, 43 SM, 44 UM-PE, and 39 UM-LE offspring were measured. HI values conformed to parametric assumptions as determined by visual inspection of histograms and normality plot, and were compared with the “anova” function in the “nlme” R package. *Post hoc* comparisons were made using the “glht” function in the “multcomp” R package.

#### Initial prey encounter

Food was withheld until Day 4, when individuals were gently moved from their compartments and placed in circular open-field arenas (radius = 5.9 cm, 250 mL) between 21:00 and 23:00, corresponding to peak feeding time (twilight) for this species (Quintela and Andrade, 2002). Each cuttlefish was allowed 15 min to habituate to the new environment, after which time filming commenced for 15 min (Panasonic HDC-SD60) and a single shrimp (C. crangon, total length 0.7—1.4 cm) was introduced. Videos were analyzed using VLC Media Player and ImageJ to collect data. The moment that cuttlefish orientated toward shrimp with their body was defined as the “time of detection” while the moment that tentacles touched the shrimp and subdued it successfully was defined as the “time of capture.” Most caught shrimp on the first attempt, although any tentacle extensions without successful capture of the shrimp were recorded as a “failed capture attempt.” Seven variables were calculated from this information: latency to detection (time between prey introduction and detection), latency to attack (time between detection and first strike at prey), latency to capture (time between detection and capture), distance of detection (distance between nearest cuttlefish eye and shrimp at time of detection), attempted capture rate (percentage of cuttlefish that attempted capture), capture rate (percentage of cuttlefish attempting capture that succeeded in capturing the shrimp) and success rate (percentage of attempted captures that were successful). In total, 56 WM, 37 UM-C, 40 SM, 38 UM-PE, and 42 UM-LE offspring were tested. Latencies and distance of detection did not meet parametric assumptions, so groups were compared with exact Kruskal-Wallis tests by Monte Carlo sampling followed by *post hoc* exact permutation tests (with sequential Bonferroni correction). The variables “attempted capture rate,” “capture rate,” and “success rate” were compared with chi square exact tests.

#### Visual laterality test

These tests were conducted between 10:00 and 22:00 5 days after hatching. The testing apparatus consisted of a start box (3.5 × 5 cm), a movable transparent barrier and two darkened shelters (3.5 × 4 cm) located 15 cm apart (see Jozet-Alves et al., 2012). Each shelter contained blue aquarium gravel and was shaded with a plastic cover. The apparatus was filled with seawater (renewed between trials) and placed under a bright fluorescent lamp (5.5 lux at the surface of the arena). In order to determine if stress induced a population-level eye-use preference, individuals were tested for shelter choice (in randomized order) by gently positioning them in the start box in such a way that it could view both shelters. Once the cuttlefish was in position, the transparent barrier was removed and the cuttlefish was allowed free access to the entire arena. Bright light is unpleasant to cuttlefish, and thus they were highly motivated to exit the start box and seek one of the darkened shelters. In total, 43 WM, 40 UM-C, 43 SM, 42 UM-PE, and 41 UM-LE offspring were tested. Within-group comparisons (the proportion turning right vs. left) were made with binomial tests and between-group comparisons (whether the proportion of those turning left differed between maternal or embryonic stress groups) were analyzed with chi square exact tests.

#### Overnight activity analysis

At midnight of Day 9, four cuttlefish from each daily cohort were randomly selected and placed in a circular open-field arena (radius = 5.9 cm, depth = 2.3 cm, 250 mL) made of opaque white plastic (sides) and a glass base. Illuminated from below by infrared light (which is not visible to the cuttlefish but is recorded by the camera), each individual was filmed from overhead for 6 h with a software-specific camera in a darkened room. This period corresponds with the times at which cuttlefish have been found to be most active (Denton and Gilpin-Brown, 1961; Jäckel et al., 2007; Frank et al., 2012; Oliveira et al., 2017). Videos were analyzed with Ethovision (Noldus®), a software package for behavioral tracking. The total distance traveled, time spent moving, and mean meander were recorded for each individual. Some individuals were unusable due to poor lighting and were excluded. In total, 20 WM, 10 UM-C, 15 SM, 8 UM-PE and eight UM-LE offspring were analyzed. These data did not conform to parametric assumptions, so were analyzed with exact Kruskal-Wallis tests followed by *post hoc* exact permutation tests (sequential Bonferroni correction).

#### Threat response activity analysis

At noon on Day 7, two pairs of treatment- and age-matched cuttlefish were randomly selected from the daily cohort. They were placed in the open-field arena described in the previous paragraph and recorded and tracked in the same manner. After 1 h of filming, 50 ml of “blank” water from the UM-C egg tank was added to the arena of one member of each pair and 50 ml of “predator odor” water from the UM-PE egg tank containing the three seabass (*D. labrax*) was added to their counterparts' arenas. This was accomplished using tubes already present beneath the waterline of each arena in order to minimize the disturbance of the addition of water. The total distance traveled and time spent moving were recorded for each individual in the same manner as described above. To control for individual differences, post treatment values are expressed as a percentage of the initial hour for each individual (baseline). In total, groups of 10 UM-C, SM, UM-PE, and UM-LE offspring were divided into “blank” (*n* = 5 per stress group) and “predator odor” treatments (*n* = 5 per stress group). These data did not conform to parametric assumptions, so were analyzed with a non-parametric analysis of longitudinal data (R package “nparLD”) followed by *post hoc* exact permutation tests (sequential Bonferroni correction).

### Ethical note

This research followed the guidance given by Directive 2010/63/EU, and French regulations regarding the use of animals for experimental procedures, and was approved by the Regional Ethical Committee Cenomexa (Committee agreement number: 54; project agreement number: A14384001). The experiment was designed to decrease animal distress by minimizing the number of animals. Enrichment was provided to unstressed captive adult cuttlefish. After spawning, adult females died naturally following senescence (June/July). After the completion of behavioral experiments, juvenile cuttlefish were anesthetized in 17.5g/L MgCl2 and euthanized with an overdose of ethanol (2%) for neurobiological testing (results not detailed here).

## Results

### Body patterning

**Figure 3:**
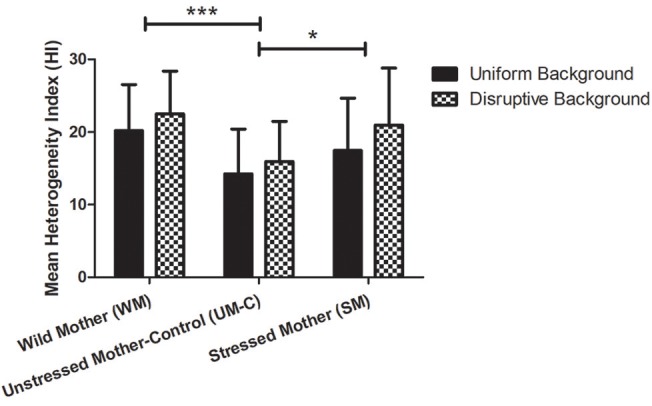
Heterogeneity Index (HI) ± s.d. of maternal stress groups on uniform and disruptive backgrounds. Between groups, WM offspring (*n* = 55) and SM (*n* = 43) had significantly higher HI than UM-C (*n* = 41; *p* < 0.001 and = 0.034). Significant differences between groups are indicated by connecting brackets. ^*^p < 0.05; ^***^p < 0.001.

In the maternal stress groups, a repeated measures ANOVA revealed a significant effect of the background type (i.e., uniform vs. disruptive: *p* = 0.001; *F* = 11.299), and of the treatment groups (*p* < 0.001; *F* = 15.66). As no interaction was found (*p* = 0.915; *F* = 0.089), this analysis showed that mean HI are higher on the disruptive background whatever the group considered (Figure 3). Pairwise *post hoc* comparisons showed that mean HI values are lower in UM-C eggs than in WM eggs (*p* < 0.001) and SM eggs (*p* = 0.034). There was no significant difference between WM and SM HI scores (*p* = 0.021).

In the embryonic stress groups, a repeated measures ANOVA revealed a significant effect of the background type (i.e., uniform vs. disruptive: *p* = 0.007; *F* = 7.493), but not of stress treatment groups (*p* = 0.066; *F* = 2.733). As no interaction was found (*p* = 0.893), this analysis indicates that mean HIs are higher on the disruptive background in all groups (data not shown).

### Initial prey encounter

In the maternal stress groups, there were no significant differences between groups for any of the variables measured (data not included).

Among the embryonic stress groups, there were no significant differences between groups in latency of detection, latency to attack, latency to capture or success rate (data not included). However, distance of detection was significantly different among the treatment groups (exact Kruskal-Wallis test: *p* = 0.0178; *H* = 7.636). Pairwise *post hoc* tests showed that this distance was significantly lower in UM-PE than in UM-LE (exact permutation test, sequential Bonferroni correction: *p* = 0.008; see Table 1). Attempted capture rate was also significantly different among the treatment groups (chi-square test: *p* < 0.001; *X*^2^ = 18.795). Pairwise *post hoc T*-tests showed that this rate was higher in UM-LE than in UM-C and UM-PE groups (Table 1).

**Table 1:** Attempted capture rate (percentage of cuttlefish that attempted captured), capture rate (percentage of cuttlefish that captured shrimp), success rate (the percentage of successful captures) of embryonic stress groups during the initial prey encounter.

	**UM-Control *n* = 35**	**UM-Predator Exposed (natural stressor) *n* = 37**	**UM-Light Exposed (artificial stressor) *n* = 34**	**Group comparisons**	***Post hoc* tests**
Attempted capture rate (%)	40.0	48.65	88.24	*p* < 0.001, *X*^2^ = 18.795	UM-C vs. UM-LE: *p* < 0.001 UM-LE vs. UM-PE: *p* = 0.008
Capture rate (%)	85.71	88.89	96.67	*p* = 0.492, *X*^2^ = 1.862	
Success rate (%)	85.71	84.21	96.67	*p* = 0.333, *X*^2^ = 2.568	

*Both group comparisons and post hocs are chi squared exact tests (sequential Bonferroni correction)*.

### Visual laterality test

**Figure 4:**
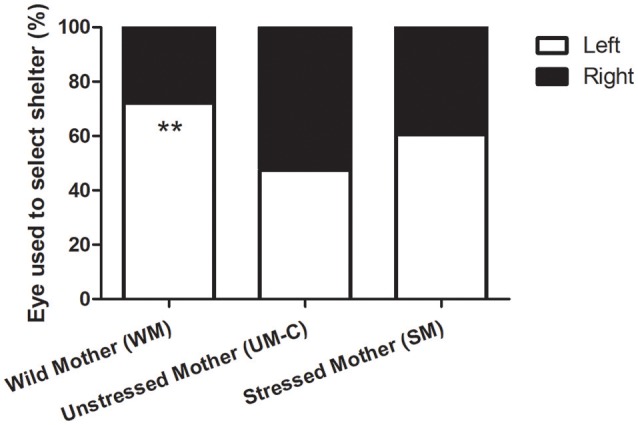
Eye used to select shelter in maternal stress groups. More WM (*n* = 43) chose the shelter in their left visual field (binomial test; *p* = 0.005, signified by asterisks) while no preference was found in UM-C (*n* = 40) or SM (*n* = 43). The proportions were not significantly different between groups (*p* = 0.08).

In the maternal stress groups, 72.1% of WM (*n* = 43), 47.5% of UM-C (*n* = 40) and 60.5% of SM (*n* = 43) offspring chose the shelter viewed in their left visual field (Figure 4). This group-level bias was only significant in WM group (exact binomial tests: *p* = 0.005). The proportion of individuals choosing the shelter located in their left or their right visual field was not significantly different between groups (chi square exact test: *p* = 0.083; *X*^2^ = 5.237).

In the embryonic stress groups, 47.5% of UM-C (*n* = 40), 59.5% of UM-PE (*n* = 42) and 61.0% of UM-LE (*n* = 41) offspring chose the shelter perceived in their left visual field (data not included). No group-level bias was found, whatever the group considered (binomial tests). The proportion of individuals choosing the shelter located in their left or their right visual field was not significantly different between groups (chi square exact test: *p* = 0.434; *X*^2^ = 1.797).

### Overnight activity analysis

**Figure 5:**
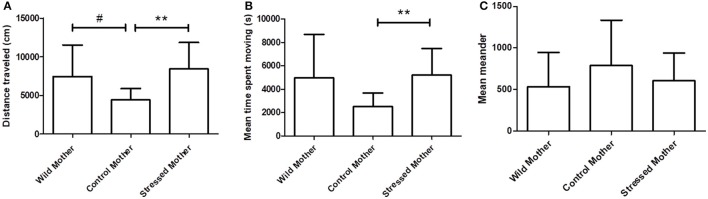
The total distance traveled **(A)**, time spent moving **(B)** and mean meander (turn angle/distance traveled; **(C)** ± s.d. of maternal stress groups in the overnight activity analysis test. Significant differences (indicated by connecting brackets with asterisks) exist between UM-C (*n* = 10) and SM (*n* = 15) in both distance traveled and time spent moving (*p* = 0.009 and 0.005; *post hoc* asymptotic permutation tests with sequential Bonferroni correction. WM *n* = 20. ^**^p < 0.01; ^#^indicates a statistical tendency (*p* < 0.08).

In the maternal stress groups, the distance traveled and time spent moving (Figures 5A,B) were significantly different between groups (Kruskal-Wallis tests: distance: *p* = 0.009; *H* = 8.982; time moving: *p* = 0.028; *H* = 7.036). Pairwise *post hoc* comparisons showed that both variables were significantly greater in SM (*n* = 15) than in UM-C offspring (*n* = 10) (exact permutation tests: distance: *p* = 0.002; time: *p* = 0.005). Finally, no significant differences existed between groups in mean meander (Kruskal-Wallis test: *p* = 0.374; *H* = 1.965; Figure 5C). In addition, WM showed a statistical trend for higher distance traveled than UM-C (exact permutation tests: *p* = 0.058).

In the embryonic stress groups, there were no significant differences between groups for any of the variables measured (Kruskal-Wallis tests; data not included).

### Threat response activity analysis

**Figure 6:**
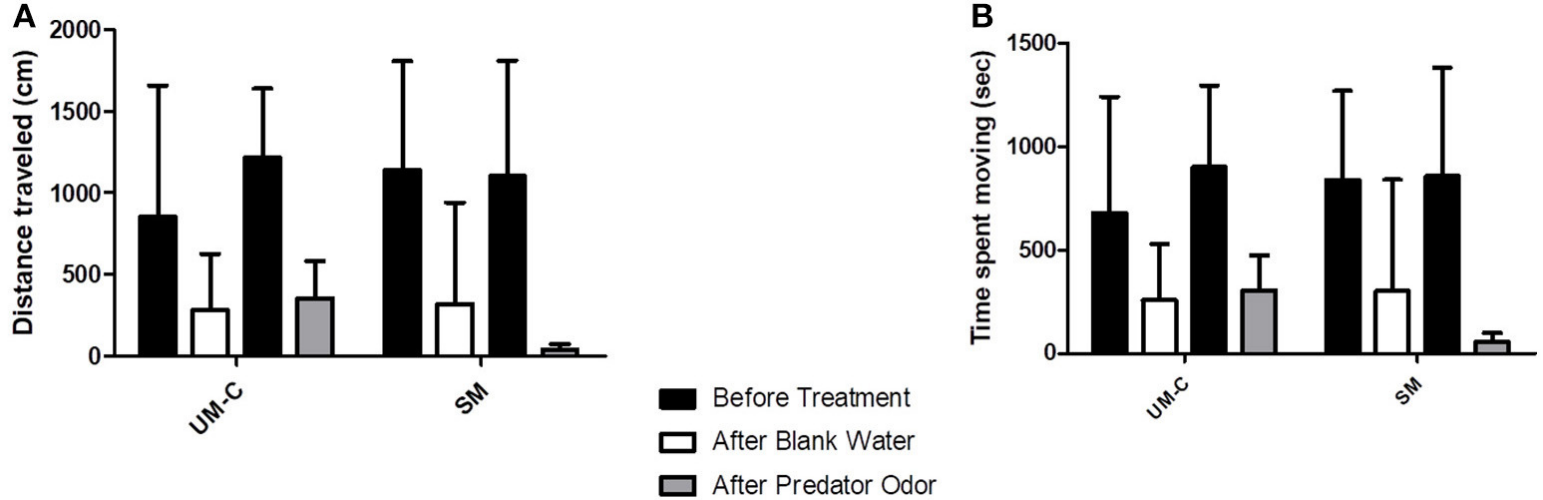
The total distance traveled **(A)** and time moving **(B)** ± s.d. for maternal stress groups in the threat response activity analysis. Differences within groups are indicated by connecting bars; *n* = 5 for all bars.

In the maternal stress groups, the non-parametric analysis for longitudinal data revealed a significant difference within groups according to time (i.e., before vs. after water addition), but not according to treatment groups (i.e., WM, SM, and UM-C) or cue type (i.e., blank water vs. predator odor), for both distance traveled (*p* < 0.001; *F* = 32.666; Figure 6A) and time moving (*p* < 0.001; *F* = 25.284; Figure 6B). As no interaction was found, this analysis showed that mean distance traveled and time spent moving are decreasing after adding water whatever the treatment group and the cue type considered.

**Figure 7:**
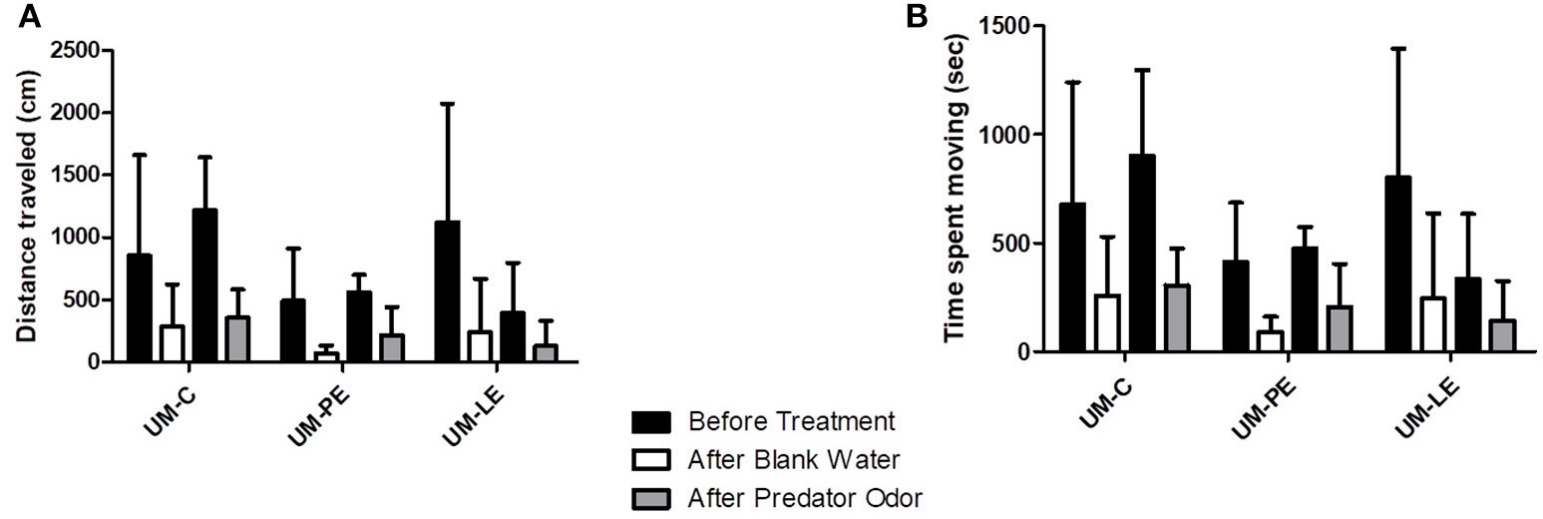
The total distance traveled **(A)** and time moving **(B)** ± s.d. for embryonic stress groups in the threat response activity analysis. Differences within groups are indicated by connecting bars; *n* = 5 for all bars.

In the embryonic stress groups, the non-parametric analysis for longitudinal data revealed a significant difference within groups according to time (i.e., before vs. after water addition), but not according to treatment groups (i.e., UM-C, UM-PE, and UM-LE) or cue type (i.e., blank water vs. predator odor), for both distance traveled (*p* < 0.001; *F* = 37.982; Figure 7A) and time moving (*p* < 0.001; *F* = 32.437; Figure 7B). As no interaction was found, this analysis showed that mean distance traveled and time spent moving decrease after adding water whatever the treatment group and the cue type considered.

## Discussion

We conducted this experiment with the aim of determining if prenatal stress affects cuttlefish behavior, and to compare various stressor types. We found that maternal stress was associated with differences in offspring body patterning and activity patterns. By contrast, offspring exposed to a natural stressor, predator odor, showed no differences from controls, while embryos exposed to an artificial stressor, bright light, differed in their predation behavior. In addition, we found that maternal captivity during spawning may affect visual laterality (summarized in Table 2).

**Table 2:** Summary of behavioral test results in comparison to the unstressed control mothers.

	**Body Patterning**	**Predatory Behavior**	**Visual Laterality**	**Activity Patterns**	**Threat Response**
	**2015 data**	**2015 data**	**2015 data**	**2015 data**	**2016 data**
Wild Mother offspring (WM)	Higher disruptiveness	No effect	Group-level left bias not observed in control group	Statistical tendency for higher distance traveled	Not tested
Stressed Mother offspring (SM)	Higher disruptiveness	No effect	No effect	Greater distance traveled and time spent moving	No effect
Natural stressor: Predator-exposed as eggs (UM-PE)	No effect	No effect	No effect	No effect	No effect
Artificial stressor: Light exposed as eggs (UM-LE)	No effect	Higher attempted capture rate	No effect	No effect	No effect

### Body patterning

In all groups, the mean HI (disruptiveness) on the disruptive background was consistently higher than that of the uniform one, suggesting that all cuttlefish adjusted their body patterns to the background. Significant differences were also seen between groups: In our experiment, maternal stress increased the mean disruptiveness of the body pattern displayed. Our results also suggest that female captivity during egg-laying can induce a group bias for higher disruptiveness in her offspring, since the offspring of wild mothers had the highest HI overall. Previous experiments with cuttlefish hatchlings have detected similar differences in body patterning between groups incubated in different environments (O'Brien et al., 2016a) and exposed to certain pharmaceuticals during development (Di Poi et al., 2014; Bidel et al., 2016). The existence of similar differences between maternal stress groups in this experiment indicates that maternal experience can also affect this behavior, and may be adaptive for their offspring—higher disruptiveness could potentially improve camouflage on the variegated backgrounds often present in the natural environment.

Where the tactic of adult cuttlefish is often to match the background by expressing more uniform patterns in response to uniform backgrounds and more disruptive patterns in response to disruptive ones (Mathger et al., 2007; Barbosa et al., 2008), young cuttlefish usually display a fairly chronic body pattern that often clashes with the background (Hanlon and Messenger, 1988; Poirier et al., 2005). The ability to produce a uniform body pattern emerges during the first few months of life (see O'Brien et al., 2016b), and the results of the present experiments suggest that maternal stress and environment may delay the emergence of this ability.

### Predation

Almost twice as many UM-LE offspring attempted capture than UM-C or UM-PE. Light is known to influence the timing of hatching (Paulij et al., 1991), and it is possible that these offspring had higher feeding motivation at the same age than other hatchlings because of increased energetic needs due to accelerated embryonic development. Faster development could also have accelerated visual maturation, leading UM-LE hatchlings to be better than their siblings at detecting prey. Indeed, UM-LE were able to detect prey at a significantly greater distance than UM-PE. It is worth noting however, that although a greater proportion of UM-LE captured shrimp, they were not better predators than the other groups, since the capture and success did not differ significantly between groups (close to 100%). This is in accordance with early experiments suggesting that prey capture operates using a highly-stereotyped program that improves little with age or experience (Wells, 1958). Despite not being better at hunting, young cuttlefish with higher feeding motivation would likely grow faster from consuming more prey.

### Visual laterality

In our experiment, no group-level bias was found in the control group. This is in accordance with previous experiments showing that a left eye-use preference for shelter seeking is not fully developed until a month after hatching (Jozet-Alves et al., 2012). Among all other groups, only WM group displayed a group-level preference toward the left side on Day 5. These results do conflict somewhat with the findings of Jozet-Alves and Hebert (2012); in that study, the authors showed that prenatal exposure to predator odor induced a left preference 3 days after hatching. However, this preference was slight, and it was necessary to test each cuttlefish more than once to detect it. Our experiment used a single trial per individual, a method formerly utilized in birds (Pittet et al., 2009), and it is possible that running only one trial did not allow us to detect the presence of the fledgling eye-use preference seen in the other groups.

The fact that eye-use preference did exist in the WM group suggests that when egg-laying and early development occur in the wild, the maturation of the left eye use preference is faster. Being lateralized from hatching may have an adaptive advantage by rendering WM offspring able to dual task (Vallortigara and Rogers, 2005). For example, while using their right eye for hunting (Schnell et al., 2016) they can simultaneously “keep an eye out” for shelter with their left should the need for a rapid escape arise.

### Overnight activity

In our assessment of baseline activity level, we found no differences between embryonic stress groups, while stressed mother offspring were associated with greater activity than control mother offspring, and similar to that of WM. We also observed a statistical tendency for WM hatchlings to travel a greater distance than UM-C. Activity levels and open field behavior have been used in behavioral research as a means of quantifying the impacts of various prenatal stressors in a variety of animals. No previously-published studies have measured this behavior in cuttlefish hatchlings, but we can draw insight from other species.

Some species, including rhesus monkeys and salmon, demonstrate decreases in overall activity after maternal or embryonic stress (Schneider, 1992; Clarke et al., 1996; Espmark et al., 2008), while others, including blue foxes and Japanese quails, show increases in activity and steps taken in open field tests (Braastad, 1998; Guibert et al., 2011). The effects of prenatal stress on activity have been studied most extensively in rodents, especially rats, and results are mixed. Some authors (Masterpasqua et al., 1976; Peters, 1986; Hilakivi et al., 1989; Sandi et al., 1996; Wilson et al., 2013) report increases in exploration and open field activity. Others report no or little effect of stress (Chapman and Stern, 1979; Van den Hove et al., 2005), or even opposite effects according to sex (Alonso et al., 1991). The majority of studies however, find decreases in movement and “exploration” in the offspring of females subjected to a variety of stressors during pregnancy (Hockman, 1961; Fride et al., 1986; Suchecki and Neto, 1991; Poltyrev et al., 1996; Vallee et al., 1997; Fujioka et al., 2001; Patin et al., 2004). Thus it seems that cuttlefish may differ in this respect from most vertebrate models and could therefore serve as a means to explore the factors driving the evolution of this response in different animal groups.

Based on insight from the studies in other animals that do show activity increases (cited above), the greater activity level observed in SM may reflect a search for shelter or food or an urge to escape. This could be advantageous by allowing young cuttlefish to avoid predation and to grow more quickly. It is also worth noting that an open field test conducted under laboratory conditions may not reflect “natural” behavior that would be seen in the wild. Indeed, a study in lab mice that compared open field behavior in the lab to the same test conducted in an outdoor grassy field found marked limitation in the number of behaviors expressed in the artificial setting (Fiore et al., 1995).

The group differences observed suggests that the stress experienced by the females during egg-laying was transmitted to their offspring and altered behavioral patterns. Physiologically, such an increase might be the result of slower vertical lobe maturation. This is the area of the brain potentially responsible for behavioral inhibition (Dickel et al., 2001, 2006), and a less mature VL would permit a higher level of basal activity. This experiment provides a starting point for future activity analyses with hatchling cuttlefish.

### Threat response

Many animals strongly alter their activity patterns in response to predator odor, especially in aquatic ecosystems. In particular, there is an extensive amount of literature documenting the behavioral responses of numerous aquatic gastropod and bivalve species (the extant molluscan groups most closely related to cephalopods) to waterborne predator odors, including escape responses such as crawling out of the water or burying (e.g., Snyder and Snyder, 1971; Jacobsen and Stabell, 2004; Dalesman et al., 2006), as well as reductions in movement such as cessation of filter feeding or decreases in foraging and migration (e.g., Reimer and Tedengren, 1997; Smee and Weissburg, 2006). Adult cuttlefish are known to react to predators with increases in escape behavior (Staudinger et al., 2013) and numerous body pattering displays (Adamo et al., 2006). Cuttlefish embryos are able to detect odors starting during the final third of embryonic development and respond to it in various ways, including embryonic increases in breathing rate (Romagny et al., 2012; Mezrai et al., in preparation), as well as post-natal behavioral lateralization (Jozet-Alves and Hebert, 2012) and changes in prey preference (Guibé et al., 2010). Thus, the ability to detect waterborne predator cues is present before hatching. The existence of odor-induced anti-predator responses in other molluscs, coupled with chemosensory abilities of embryonic cuttlefish, led us to predict that a change in activity pattern would be observable in response to predator odor in young hatchlings. The predator cue we utilized came from sea bass, which are known to prey on hatchling cuttlefish in the wild (Blanc and Daguzan, 1999), and thus represent an imminent threat to survival which should elicit a change in movement.

A reduction in activity was observed in all groups after the addition of either predator odor or blank water. This was a continuation of a pattern of progressively decreasing activity over time, and no group's reaction to predator odor differed from that of their response to blank water. Thus, it seems that unlike many other molluscs and adult cuttlefish, hatchling cuttlefish do not possess a marked locomotory threat response. Perhaps they rely exclusively on burying and/or body patterning to avoid predation. Unfortunately, the video quality and lack of sand necessary for the behavioral tracking software to function optimally prevented us from observing any burying or body patterning response. Researchers should take advantage of evolving video analysis technology to incorporate these possibilities into future tests of activity and threat response.

### Maternal vs. embryonic stress

Body patterning and activity levels were both affected by maternal stress, while embryonic stress only affected one aspect of predatory behavior. Additionally, the differences between WM and UM-C in activity and turning bias suggest that the environment in which eggs are laid can also affect offspring behavior. In sum, maternal stress and spawning environment resulted in more post-natal behavioral changes than the direct experience of stressors in the egg. The greater post-natal reaction to the maternal stimuli suggests that mothers' experience might be a more reliable indicator of future prospects than stressors experienced by the embryos directly.

Maternal experience is known to “program” offspring in many other species; most commonly, the offspring of mothers exposed to a particular predator showed adaptive responses when encountering that predator itself (reviewed in Agrawal et al., 1999; Storm and Lima, 2010). In birds and mammals, such maternal stress effects are likely mediated by the transfer of stress hormones in the egg or placenta (Hayward and Wingfield, 2004; Groothuis et al., 2005; Weinstock, 2008). Since cuttlefish lack a planktonic larval phase and their dispersal abilities are likely limited by their size, any dangers present at or near the spawning site are likely to be a threat to cuttlefish at hatching. Anticipating and preparing for these threats makes adaptive sense. The higher disruptiveness and greater activity levels of stressed mother offspring and the higher disruptiveness and left turning bias of wild mothers could be advantageous to hatchlings by improving camouflage and facilitating escape from predators.

The effects of maternal environment and stress should be taken into account when planning, conducting and interpreting future laboratory experiments with cuttlefish—the behaviors observed may differ depending on how subjects were obtained (i.e., bred in captivity or collected from the wild) and handled, and experimenters should carefully consider their experimental priorities (i.e., whether they are trying to assess natural behavior) before they source cuttlefish eggs for experiments. More broadly, further experimentation in other oviparous species is important in understanding the results obtained in viviparous and ovoviviparous species for which maternal and embryonic effects cannot be disassociated.

### Artificial vs. natural embryonic stressors

Sea bass (*D. labrax*) are a particularly relevant stressor to cuttlefish since they have long co-existed in the English Channel and readily predate on hatchling and juvenile cuttlefish (Blanc and Daguzan, 1999). Sensing sea bass odor in the natal environment is a direct signal of post-natal threat for hatchling cuttlefish. Because of this, selective pressure for embryos to detect and prepare for this threat is presumably strong. Indeed, embryonic exposure to seabass odor is associated with increased lateralization in cuttlefish hatchlings, a behavioral adaptation which can facilitate rapid escape (Jozet-Alves and Hebert, 2012). In these five experiments however, the predator cues had no discernable behavioral effect.

It is possible that embryos habituated to the predator odor. In our experiment, UM-PE embryos were housed in tanks with seabass for most of development, and had the ability to sense odor cues for the last seven (of 30) stages of embryonic development (Romagny et al., 2012). Thus, they had at least several weeks of chemosensory exposure to these predators. Post-natal studies in other animals, including fish, rats and lizards, have shown that while acute stress exposure can result in adaptive changes (e.g., increased predator avoidance behavior or HPA-axis sensitivity), long-term or repeated exposure can actually reduce or eliminate the adaptive response (Dielenberg and McGregor, 1999; Weinberg et al., 2009). On the other hand, some studies show a lack of habituation to predator odor applied long-term (e.g., Epple et al., 1993). If habituation to predator odor is indeed occurring in cuttlefish, the evolutionary reason for this merits further scrutiny. One possibility is that because the predator odor was not paired with alarm cues from injured conspecifics in our experiment, the cuttlefish embryos learned to regard it as benign. Such a phenomenon occurs in harbor seals, which learn to distinguish between the calls of fish-eating and seal-eating orca populations and behave accordingly (Deecke et al., 2002).

In parallel, we tested an “artificial” stressor that could be compared to the effect of predator odor. We selected an artificial light source (LED panels) at a high intensity to penetrate the opaque egg membrane. The timing of the light regime was randomized and mimicked what might occur in some artificial settings. Though this stressor was a completely artificial stimulus and not indicative of a threat, it was associated with a strong, seemingly adaptive effect on predation behavior. Thus, our prediction of positive effects in response to predator odor and of disruptive effects in response to LED light was not supported by these results. This suggests that the evolutionary “familiarity” of a stressor (i.e., whether the species has encountered it before) is not the only explanation for fitness differences in the stressor response. The fact that we found an effect of light (increased predation) and no effect of predator odor may instead be explained by the relevance of the sensory modalities engaged by each stressor. While both odor and light can be perceived and responded to by embryos, cuttlefish are highly visual animals (Darmaillacq et al., 2017), and thus visual cues are likely to be more relevant to them than odor cues. Alternatively, this behavior may simply reflect a physiological improvement in visual acuity due to the wider ranges of light intensity experienced during embryonic development. Further testing exploring the role of different cues and sensory modalities are ongoing (Mezrai, in preparation).

## Conclusion

The results reported here can serve as a basis for future behavioral tests examining prenatal stress and other embryonic influences. The tests utilized were non-invasive methods and, when employed as a battery, cover a broad range of behaviors critical to survival that give a rough measure of offspring fitness and treatment group differences. In particular, the activity analyses and threat response test were the first to be conducted with hatchling cuttlefish, and should offer valuable baseline data for researchers hoping to utilize such tests in the future. Further experimentation with other sources of prenatal stress will elaborate on the results reported here and could reveal previously-unknown prenatal pressures driving offspring behavior.

At the same time, greater effort should be made to account for the effects of spawning environment and early stimulation when planning and interpreting laboratory experiments and in the welfare of this regulated species. It is well-established that environmental enrichment is crucial to early cognitive development in cuttlefish (Dickel et al., 2000) and is recommended for the welfare of adults (Fiorito et al., 2015). The results presented here underscore the importance of maintaining a stimulating environment for reproducing females and even potentially their eggs. Researchers should strive to maintain at least a basic level of sensory enrichment for captive adults, and carefully consider the environmental cues experienced by developing eggs. Future guidelines will hopefully standardize a basic level of enrichment for all European cuttlefish research. It may even be beneficial to include predator cues and other mild stressors to encourage the development of certain aspects of the behavioral phenotype (e.g., hunting ability). Carefully adapting captive enclosures to cuttlefish needs will ensure the psychological well-being of individuals and the reliability of experimental results, promote growth in aquaculture and yield more savvy offspring for future hatch and release programs.

## Summary statement

The effects of several chronic prenatal stressors (maternal stress, embryonic exposure to predator odor or bright light) on hatchling cuttlefish are compared in five tests.

## Author contributions

CO: Primary data collector and author of article; CJ-A: Assisted with data analysis, experimental design and editing of manuscript; NM: Data collector, editor of manuscript; CB: Data collector, editor of manuscript; A-SD: Supervisor, editor of manuscript; LD; Primary supervisor, editor of manuscript.

### Conflict of interest statement

The authors declare that the research was conducted in the absence of any commercial or financial relationships that could be construed as a potential conflict of interest.

## Abbreviations

UM-C: Unstressed Mother Control eggs
SM: Stressed Mother eggs
WM: Wild Mother eggs
UM-PE: Unstressed Mother Predator-Exposed eggs
UM-LE: Unstressed Mother Light-Exposed eggs
HI: Heterogeneity Index.
